# Fine-scale Southern California Moho structure uncovered with distributed acoustic sensing

**DOI:** 10.1126/sciadv.adr3327

**Published:** 2024-11-27

**Authors:** James Atterholt, Zhongwen Zhan

**Affiliations:** California Institute of Technology, Pasadena, CA, USA.

## Abstract

Moho topography yields insights into the evolution of the lithosphere and the strength of the lower crust. The Moho reflected phase (PmP) samples this key boundary and may be used in concert with the first arriving P phase to infer crustal thickness. The densely sampled station coverage of distributed acoustic sensing arrays allows for the observation of PmP at fine-scale intervals over many kilometers with individual events. We use PmP recorded by a 100-km-long fiber that traverses a path between Ridgecrest, CA and Barstow, CA to explore Moho variability in Southern California. With hundreds of well-recorded events, we verify that PmP is observable and develop a technique to identify and pick P-PmP differential times with high confidence. We use these observations to constrain Moho depth throughout Southern California, and we find that short-wavelength variability in crustal thickness is abundant, with sharp changes across the Garlock Fault and Coso Volcanic Field.

## Teaser

The Moho reflected phase measured by DAS shows abrupt crustal thickness changes across the Garlock Fault and Coso Volcanic Field.

## INTRODUCTION

Observations of Moho structure are important because they provide evidence of processes that deform the lithosphere and yield constraints on the rheology of the lower crust (*1*, *2*). Crustal thickness varies over many scales, and fine-scale variability (on the order of a few kilometers) is important because it constrains the depth-extent and depth-dependent behavior of localized tectonic processes observed at the surface (*3*–*6*). The most popular methods for uncovering Moho structure are receiver functions and controlled-source seismic surveys. Receiver functions, which leverage secondary phases in teleseismic data, are a powerful tool for robustly determining Moho depth (*6*–*9*), but are a low-frequency measurement that requires seismometers near the measurement points, limiting their spatial resolution and geographic coverage. Controlled-source surveys are performed by recording numerous sources with shorter transitory arrays to produce seismic reflection profiles (*10*–*12*) or by measuring source wavefields over large distances with longer arrays in so-called wide-angle refraction and reflection surveys (*13*–*17*). These surveys can produce Moho phases that can be used to uncover crustal thickness variability at impressive resolution, but they are expensive, are logistically challenging, and sometimes fail to penetrate Moho depths.

An alternative to these techniques is to use arrival times of the Moho reflected phase (PmP) in regional earthquake wavefields, which can be used to constrain Moho depth (*7*, *18*–*21*) and lower crustal velocity structure (*22*–*25*). The advantage of using this observation to constrain crustal thickness over other methods is that measurements are made passively using higher-frequency regional earthquakes and, with the right seismicity distribution, can resolve structure outside a seismic network. This technique has not been used as frequently as others because PmP is difficult to confidently identify on individual seismograms (*20*). Recent studies have found success using multi-event estimates of PmP moveout and machine learning techniques to generate expanded PmP catalogs (*7*–*18*). These techniques are not readily transferrable to or ideal for dense array datasets that generally have lower data quality but high spatial sampling. The high-frequency content of PmP observations combined with dense array datasets may allow for very high-resolution sampling of short-wavelength features near the crust-mantle boundary.

Distributed acoustic sensing (DAS) is an increasingly popular technique in seismology that transforms fiber optic cables into dense arrays of strainmeters using phase interferometry of backscattered light (*26*). This technique is powerful because it enables the deployment of extensive, dense seismic networks for long periods of time at low logistical burden. DAS is routinely used for shallow crustal imaging (*27*–*29*) and has recently been used to resolve structure in the middle crust using travel-time tomography (*30*). The dense recordings enabled by DAS facilitate short-wavelength observations over large distances, and the long deployment times allow for the passive recording of high-quality earthquake wavefields with diverse source locations. While receiver functions have been successfully computed by combining DAS data with nearby broadband data (*31*), wider adoption of deep crustal imaging techniques with DAS has been slow, because DAS has high noise levels at low frequencies (*32*). PmP is an especially promising avenue for realizing the potential of DAS for deep crustal imaging for a few reasons. First, PmP is a high-frequency phase, and DAS is most sensitive to higher-frequency wavefields. Second, the high spatial density and abundance of channels in DAS arrays allows for both spatial coherence and array-side moveout to be used to identify PmP. Third, although DAS arrays have many channels, the cable geometry often imposes a narrow geographic footprint; with PmP, Moho depth measurements may be made over a much larger area than is covered by the fiber.

Here, we introduce a method with which to identify and pick relative arrival times of secondary phases in DAS data. We apply this method to a DAS array in the Mojave Desert to measure PmP differential times and use these observations to invert for Moho depth over a wide area. We find good correspondence with previous results, and we observe short-wavelength features in the Moho across the Garlock Fault and the Coso Volcanic Field (CVF). This technique offers a promising outlook for using DAS arrays to make fine-scale observations of lithospheric structure over broad geographic areas.

## RESULTS

### Autocorrelation for secondary phase retrieval

**Fig. 1.**
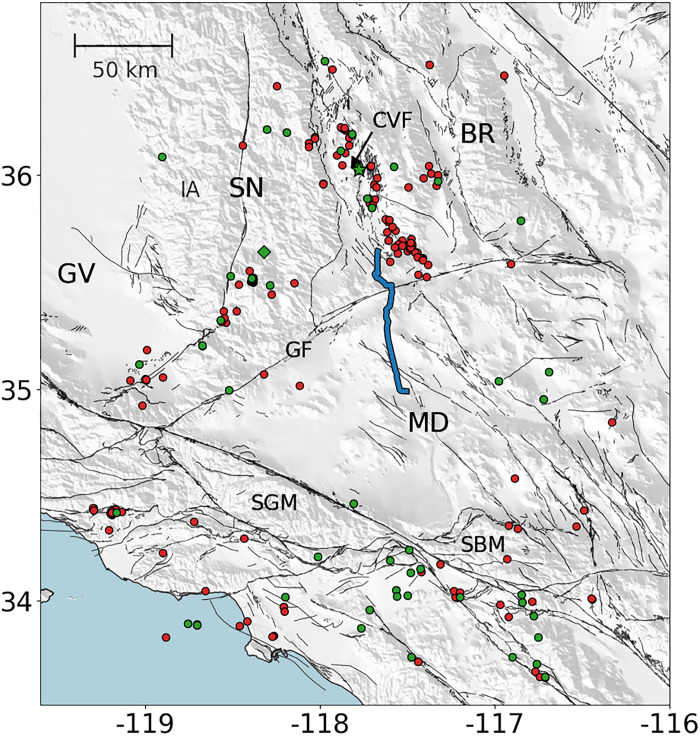
Experiment setting and data. The DAS array geometry (blue curve) plotted against the kept (green) and discarded (red) M2.5+ events recorded by the array. Also plotted are the fault traces included in the USGS Quaternary Fault Database (2018) and locations of relevant tectonic provinces and features (GV, Great Valley; SN, Sierra Nevada; BR, Basin and Range; MD, Mojave Desert; CVF, Coso Volcanic Field; GF, Garlock Fault; SGM, San Gabriel Mountains; SBM, Santa Barbara Mountains; IA, Isabella Anomaly). The green star corresponds to the event that produced the wavefield shown in Fig. 3.

**Fig. 2.**
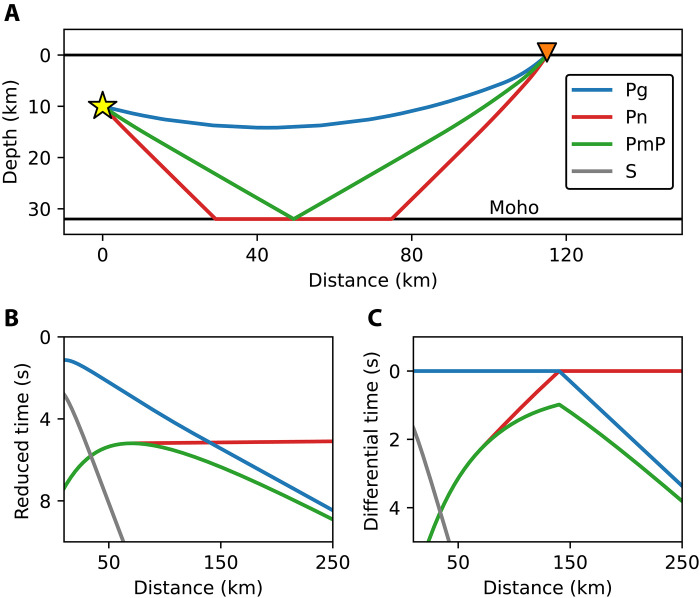
Important phases and relative arrival times. (**A**) Ray paths for the three phases of interest in this study for a fixed source-receiver distance and representative velocity model: the direct phase (Pg), the Moho head wave (Pn), and the Moho reflected wave (PmP) (S not pictured for simplicity). (**B**) Reduced (8.1 km/s) arrival times of phases plotted against source-receiver distance. (**C**) Differential times of phases relative to the first arriving P phase plotted against source-receiver distance.

In August of 2021, a DAS array was deployed on a 100-km segment of dark fiber between Ridgecrest, CA and Barstow, CA (Fig. 1). This array has 10,000 channels with 10-m channel spacing and 100-m gauge length. Over the course of 2 years, the array recorded 440 M2.5+ regional earthquakes. Many of these earthquake wavefields exhibit strong secondary phase arrivals that coincide with the expected onset time of the Moho reflected phase (PmP) for a standard earth model. The relative arrival time between the first arriving P phase and PmP provides a strong constraint on crustal thickness (Fig. 2). Coupled with the spatial density of DAS, P-PmP differential times can yield very short wavelength profiles of Moho depth.

Identifying and measuring the relative arrival times of PmP on DAS data can be a challenging problem. First, although PmP is often the strongest phase in *P*-wave coda, it has historically been very challenging to identify in broadband data, with only ~1% of records showing readily identifiable PmP waveforms (*18*). DAS presents an advantage toward identifying this phase; the spatial density of DAS arrays allows for the evaluation of candidate phases across a broad spatial window. This spatial window allows for the use of spatial coherence and array-side moveout to assist in identifying secondary phases like PmP. Once identified, however, it is impractical to pick the thousands of phase arrivals recorded by DAS for every earthquake wavefield by hand. Traditional automated phase picking methods are usually inadequate for these data because P-phase onsets are often weak and complicated in DAS due to broadside insensitivity of axial strainmeters and strong surface wave scattering due to local heterogeneities (*33*). Machine learning methods, such as PhaseNet DAS (*34*), have shown promise toward addressing this problem, but as of now, they have not been adapted to secondary phases.

**Fig. 3.**
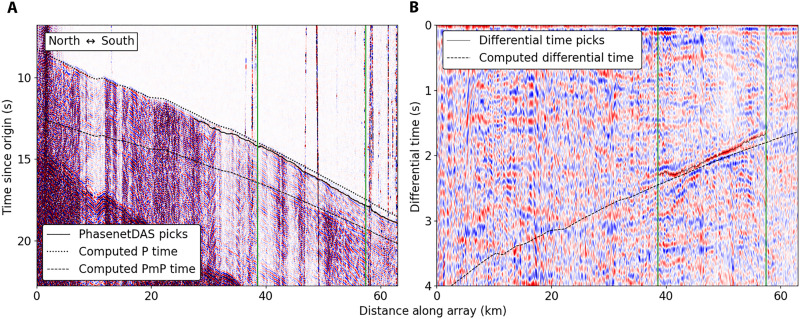
Example of autocorrelation for phase retrieval. (**A**) Earthquake wavefield with representative data quality. Included are the PhaseNet DAS picks and computed P and PmP times for the representative velocity model used in this study with a Moho depth of 32 km. Green bars are a reference for the channel bounds within which differential time picks were made. (**B**) Autocorrelation image created for the corresponding earthquake wavefield using the framework outlined in the text. Included are the computed P-PmP differential times and the picks made on the positive correlation peak associated with the PmP arrival. Location of this event is indicated as a green star in Fig. 1.

**Fig. 4.**
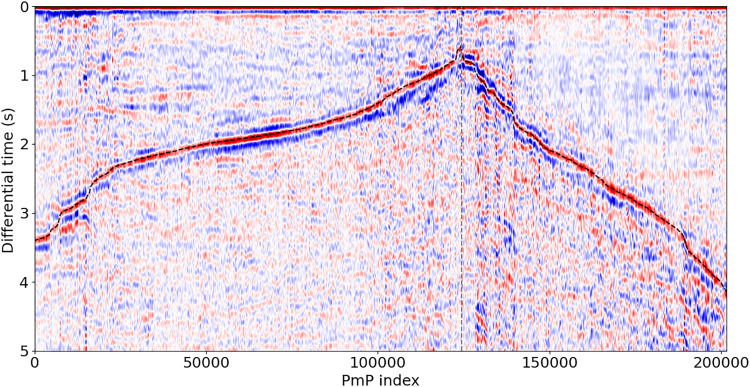
Summary of all PmP picks made in this study. Plotted are the autocorrelation correlograms for each channel for which estimates of P-PmP differential time could be made. Black dotted lines correspond to pick times, and correlograms are organized by decreasing pick times when following Pg and increasing pick times when following Pn. Gray dotted line marks the transition from Pg to Pn as first arrival.

We develop a simple and effective approach to both identify and pick relative arrival times of secondary phases like PmP. Because the scattered surface waves that are nearly ubiquitous in DAS data are generated locally (*33*–*35*), these waves are common to both the first arrival and secondary arrivals. These phases consequently generate complicated but highly correlated wavefields. The autocorrelation of DAS-recorded earthquake wavefields is thus a potentially powerful tool for making highly accurate relative phase arrival time measurements. We apply a straightforward and semi-automated framework for identifying PmP and obtaining P-to-PmP differential times for DAS data (see Materials and Methods). This methodology is outlined using an example in Fig. 3. Of the initial set of events, 229 events had visually identifiable P-phase onsets and some high-quality first arrival picks. Of this smaller subset of events, we can observe and pick PmP arrivals over large spatial windows for 72 events. Additionally, as shown in Fig. 2, by evaluating the change in differential time with source-to-receiver distance, we determine for each observation whether PmP is trailing a direct wave (Pg) or a mantle head wave (Pn) first arrival. From this dataset, our workflow yields over 200,000 accurate and precise P-PmP differential times that may be used to constrain the velocity structure and crustal thickness throughout Southern California. These picks and the corresponding correlograms are shown in Fig. 4.

### Fine-scale crustal thickness variability from dense Moho depth profiles

As shown in Fig. 2, the differential time between Pg and PmP is a function of both the difference in path length between these two phases, which is dependent on the Moho depth, and the difference in the velocity structure to which these two phases are sensitive. Pn has a longer ray path than PmP, but it arrives earlier than PmP and eventually overtakes Pg because the upper mantle velocity is much higher than the crustal velocity. When using P-PmP differential times, we thus need to consider three things: crustal structure, upper mantle velocity, and crustal thickness. Crustal structure and thickness trade off with each other, and considering the two simultaneously would result in non-uniqueness, particularly because parameterizing the crust requires both seismic velocities and a layering structure. Short-wavelength variability in PmP differential times is much more sensitive to changes in crustal thickness. This is because sensitivity due to crustal structure depends on integrated velocities along the ray path, not detailed velocity structure; we subsequently illustrate this with synthetic tests. Since we are most interested in sharp changes in the Moho, we choose to fix a representative crustal model using an ensemble of velocity profiles (see Materials and Methods) and invert for Moho topography. The distance at which Pn supersedes Pg as the first arrival and the rate at which the Pn-PmP differential time increases with distance are functions of the upper mantle velocity integrated along the ray path. While this value also trades off with absolute Moho depth, we can resolve a robust estimate of a representative upper mantle velocity for the entire dataset in a straightforward way. We can then incorporate this velocity into our model when inverting for crustal thickness.

**Fig. 5.**
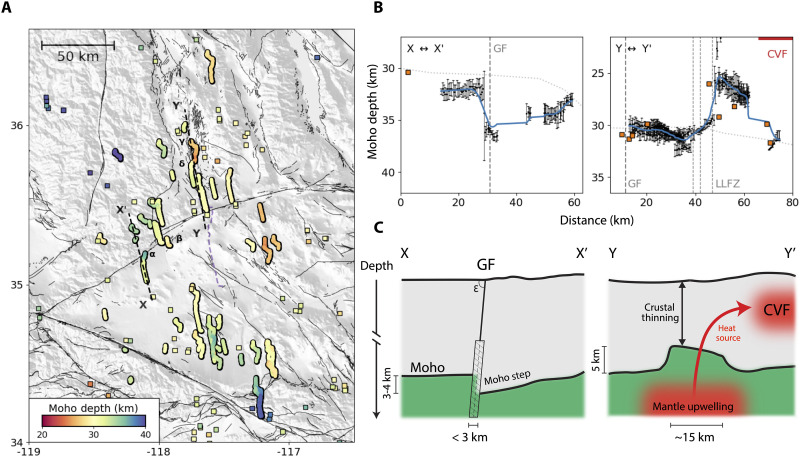
Summary of resolved Moho depth variability. (**A**) Moho depth inversion results along corresponding bounce-point locations for all events included in this study. Included as squares are Moho depth estimates reported in previous study (*9*). Purple dotted line marks the DAS array location. (**B**) Cross sections of Moho depth corresponding to the profiles mapped in (A). Included are all estimates of Moho depth within 10 km of the profile reported in this study (black dots) and all estimates of Moho depth within 20 km of the profile reported in (*9*) (orange squares). Lettering indicates different structural features where they cross the profiles (LLFZ, Little Lake Fault Zone). The CVF location marks the approximate southern extent of the mid-crustal low-velocity zone reported in (*55*). Lateral dotted line corresponds to smoothed Moho depth estimates from the Community Moho Model (*38*). (**C**) Schematic diagrams of the cross sections in (B).

Using a representative crustal model, we determine a best-fitting combination of Moho depth and upper mantle velocity for our entire dataset (see Materials and Methods). We find that the optimal pair is a Moho depth of 31.5 km and an upper mantle velocity of 8.1 km/s. The ensemble of crustal models is shown in fig. S1. This Moho depth is very similar to that of the Hadley-Kanamori (HK) model for Southern California (32 km) (*36*), and the upper mantle velocity is consistent with Pn tomography results from this region (*20*, *37*). We then use our fixed crustal and optimal upper mantle velocities combined with our P-PmP differential times and first arrival classifications to invert for crustal thickness along each of our bounce-point profiles (see Materials and Methods). The resultant profiles provide estimates of Moho depth at sub-kilometer intervals, which is smaller than the minimum wavelength expected in the earthquake wavefields used in this experiment. These Moho depth estimates and select cross sections are shown in Fig. 5.

These profiles yield Moho topography that is in close agreement with previously resolved long-wavelength features in Southern California (*38*) and provide remarkably high spatial frequency resolution along profiles that illuminate highly localized changes in crustal thickness. Broadly, we observe a Moho depth of approximately 30 km throughout most of the Mojave block that shallows to the east. We also resolve a deep Moho over and around the Isabella anomaly (*39*) that has been regularly observed in this region (*40*). Consistent with other studies, the crust thickens sharply at the transition to the San Bernadino Mountains, corresponding to the mountain root (*11*, *40*). The Moho is relatively deep just to the northwest of these mountains, which is consistent with some recent results (*7*). In general, the absolute values of Moho depth across the region agree with previous localized measurements of Moho depth made using receiver functions (*9*), as is illustrated in Fig. 5 and fig. S2.

**Fig. 6.**
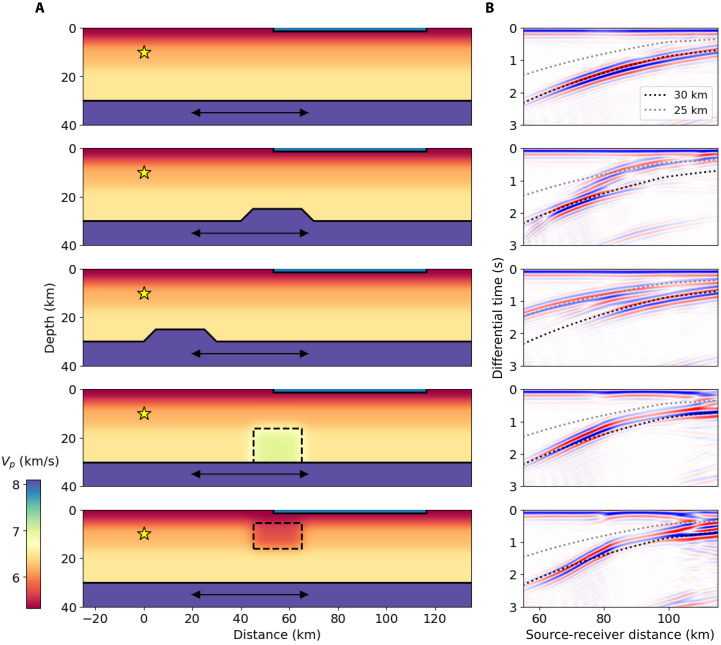
Synthetic tests. (**A**) Velocity models and source-array geometry (shown as blue line at surface) used in these synthetic tests. Double-sided arrows correspond to the approximate extent and locations of bounce points in these tests. Solid black line marks the Moho, and dotted black lines outline velocity anomalies. (**B**) Differential times from autocorrelation wavefields for each synthetic test. Shown are reference relative arrival times of PmP for a flat Moho at 25 and 30 km depth. Black arrow indicates the diffracted phase.

There are trade-offs between variability in the velocity model and crustal thickness, and we evaluate and discuss these trade-offs later. However, the primary advantage of combining a high-frequency measurement like P-PmP differential times and the high spatial sampling of DAS is toward resolving fine-scale variability in crustal thickness, rather than broad-scale absolute values. Velocity model variability will generally exert a longer wavelength effect on P-PmP differential times because the velocity contribution to the differential time is path integrated. Crustal thickness variability, however, can exert shorter wavelength effects on P-PmP differential times, because a sharp change in the Moho depth abruptly changes the path length of PmP. We demonstrate this using a set of synthetic tests (see Materials and Methods) shown in Fig. 6. These tests show that even very sharp and high-amplitude local velocity perturbations have a smooth and muted effect on P-PmP differential time with source-to-receiver distance. Comparatively, a Moho step produces an abrupt jump in the P-PmP differential time that is representative of the amplitude of the step in the Moho.

### Sharp Moho changes across the Garlock Fault and CVF

**Fig. 7.**
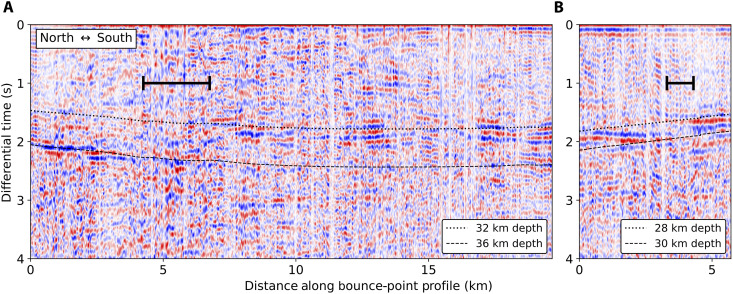
Auto-correlograms across Garlock Fault. (**A** and **B**) Correlograms for long and short bounce-point profiles across the Garlock Fault, respectively. Plotted are reference curves of expected P-PmP differential times for different Moho depths. Also plotted are the estimates of the width of uncertain Moho depths on either side of which we can confidently identify distinct PmP peaks. Locations of these profiles are indicated as α and β in Fig. 5.

**Fig. 8.**
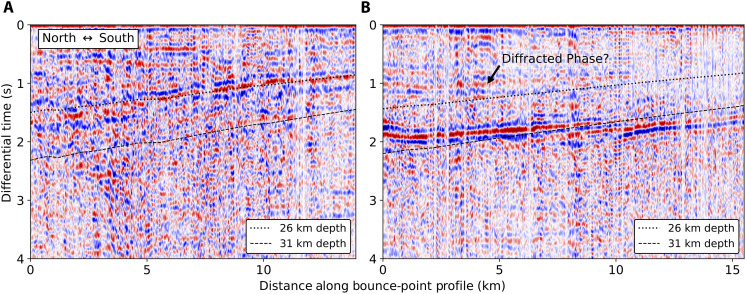
Auto-correlograms near CVF. (**A** and **B**) Correlograms for bounce-point profiles on and just south of the shallow Moho depth anomaly near Coso, respectively. Plotted are reference curves of expected P-PmP differential times for different Moho depths. Also identified is a correlogram peak that we speculate is the diffracted phase off nearby shallow Moho anomaly. Locations of these profiles are indicated as γ and δ in Fig. 5.

**Fig. 9.**
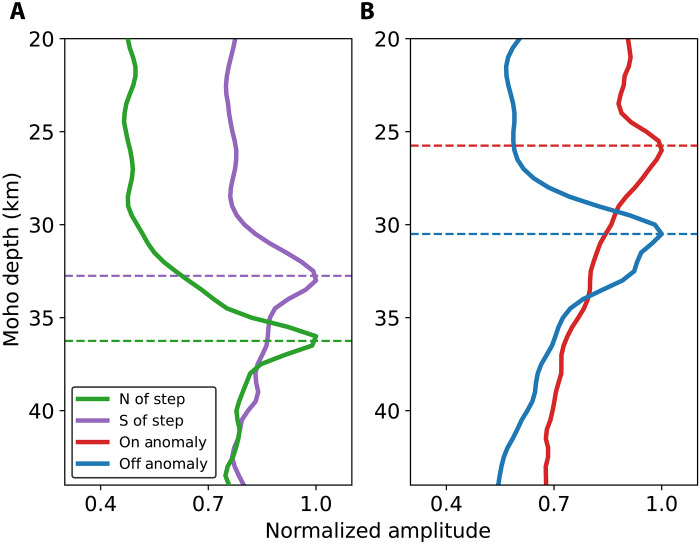
Correlogram envelope summation results. (**A**) and (**B**) show profiles that summarize the amplitude of the summation of the envelopes of the correlogram profiles shown in Figs. 7 and 8 along expected P-PmP differential times for different Moho depths. (A) shows the results of this operation performed along the spatial windows to the north and south of the expected step location for profile α shown in Fig. 7. (B) shows the results of this operation performed on profiles γ (on anomaly) and δ (off anomaly). Both (A) and (B) suggest that the data between profiles are consistent with variant crustal thickness values.

Our results show that there is considerable variation in Moho depth along our crustal thickness profiles. We choose to focus on two short-wavelength features that are observable only because of the unique combination of the station density of DAS and the regional sensitivity of the PmP measurements. The correlogram profiles that inform these observations are shown in Figs. 7 and 8. One of these interesting features shown in Fig. 5 is a sharp step (3 to 4 km) in the Moho that is very close to the surface trace of the western segment of the Garlock Fault. As shown in Fig. 7, this step is clear in the data and occurs over only a few kilometers. Because this profile is recovered using individual event wavefields, uncertainties related to source location do not affect the relative change. A less precise but more objective way to confirm the presence of the step is to sum the envelope of these correlograms along the expected P-PmP differential times for a range of Moho depths. When we do this along the spatial windows to the north and south of the expected step location, we find that there are peaks at distinct Moho depths (Fig. 9). Another shorter profile to the east, also shown in Fig. 7, shows a step-like feature of the same polarity, providing additional evidence for this Moho discontinuity.

This observation may be attributed to a discontinuity in the crust imposed by the Garlock Fault and would suggest that the western segment of the fault penetrates the Moho. This observation is distinct from some early results suggesting that the Garlock Fault is truncated (*10*) or approaches a horizontal angle in the middle crust (*41*). The results presented in this study have deeper penetration depths and require fewer assumptions than these earlier studies. The deeper penetration depths resolved in this study add increased importance to the Garlock Fault as a physical boundary between the Mojave and the Sierra Nevada and Basin and Range terrains to the north (Fig. 1). This agrees with some geologic studies that suggest the Garlock Fault delineates, and slip on the fault may be driven by, a difference in extensional behavior between the Basin and Range and the Mojave (*42*). Additionally, this feature suggests that along this segment of the fault the Garlock is nearly vertical through the entire crust. This extends the results of earlier studies that used focal mechanisms (*43*) and imaging techniques (*44*, *45*) to infer a near-vertical dipping fault at seismogenic depths. The width of this step is intriguing, because the behavior of continental faults at depth can shed light on the strength of the lower crust and upper mantle (*6*, *46*). Since the Garlock Fault consists of a narrow step, this observation supports the possibility of a narrow shear zone at depth, rather than a broad deformational zone. The wavelength of this step observed at the Garlock Fault (<3 km) suggests an upper bound for the width of the deformation zone at the crust-mantle boundary. The PmP observation becomes faint in the narrow spatial interval at the step, and we cannot further characterize the properties of the step within this interval. That such a narrow zone with steep offset is maintained on a relatively slow slipping fault (*47*) may help constrain the strength contrast at the crust mantle boundary. However, future study is required to quantify the rheological conditions necessary to preserve this step. This observation is comparable to that of the Denali fault, another intracontinental strike-slip fault with a sharp Moho step at depth (*48*). A schematic representation of the observations made at the Garlock is shown in Fig. 5C.

Another important feature is the thinning of the crust near the CVF that is illuminated by several profiles. Two of the correlograms that inform this result are shown in Fig. 8. Again, we can confirm the distinction between these profiles by summing the envelopes of the correlograms for these two profiles along computed P-PmP differential times (Fig. 9). We find that this confirms that the P-PmP differential times evidence distinct Moho depths at each profile. This observation is supported by a resolved shallowing of the Moho observed with receiver functions nearby (*9*). The crustal thinning may be explained as a highly localized feature that evidences a regional tectonic phenomenon. The deep Moho to the west, which is also resolved in our data, has been explained as a subduction-induced lithospheric drip of the ultramafic base of the Sierra Nevada batholith (*49*–*50*) or as a fragment of the Farallon Plate (*51*, *52*). In either case, these structures are expected to induce a counterflow that results in mantle upwelling and crustal thinning to the east (*53*). There are several recent results that suggest that the CVF is collocated with a notable low-velocity anomaly in the upper mantle and lower crust that are slightly to the south of the associated mid-crustal magma reservoir (*24*, *52*, *54*). Our results may provide additional constraints at the crust-mantle boundary on the connectivity between the mid-crustal magma reservoir and a mantle source that feeds the system. The feature we resolve is more local and biased further to the north than the features described in deeper tomography studies, which may be due to either distinct responses of velocity structure and Moho depth to the thermal anomaly or the different sensitivities of these methods. A schematic representation of the connection between this observed section of thinned crust and the Coso system is shown in Fig. 5C.

The known low-velocity anomaly in the middle crust associated with this reservoir (*55*) raises questions as to whether we are observing a true crustal thickness anomaly or an apparent thickness anomaly due to sharp velocity heterogeneity. A low-velocity anomaly in the lower crust would make the Moho apparently deeper, rather than shallower, but a low-velocity anomaly in the middle crust could preferentially delay Pg and produce an apparent thinning of the crust. But, as shown in our synthetics in Fig. 6, even a high-amplitude low-velocity anomaly in the middle crust, one much stronger than what is resolved in tomographic studies (*55*) and comparable to the volume expected at CVF, produces a small perturbation in P-PmP times relative to a 5-km-high Moho platform. Additionally, the synthetics show a diffracted phase generated at the transition to bounce points off the step that may be observable in the data. We observe a weak phase preceding the PmP phase in the autocorrelation profile just south of the shallow Moho anomaly; our preferred interpretation is that this is the diffracted phase (Fig. 8).

## DISCUSSION

### Ambiguity and uncertainty

There will always be uncertainty when attempting to classify secondary phases; our objective is to minimize this uncertainty. It is thus important to evaluate the confidence with which we can ascribe the secondary phase arrivals made in this study to a specific phase. There are several reasons why we expect our observations to correspond to PmP. Each identification was made using prominent phases recorded by at least hundreds of channels that are highly correlated with the first arriving P phase. This would suggest that we are not identifying spurious arrivals, but rather phases associated with a sharp and coherent discontinuity with a similar scattering behavior to the P phase. It is well documented that PmP is often the most prominent phase in the *P*-wave coda (*20*, *56*), and so we would expect it to show a high relative amplitude to the first arriving P phase. We only identified a phase as PmP if the phase approximately paralleled the expected P-PmP differential time and moveout for a representative lithospheric model of the region. This correspondence was usually evaluated over tens of kilometers at meter-scale intervals for each event, as shown in Fig. 3. This array-side moveout evaluation is an effective tool for identifying secondary phases that is available only to dense arrays. Other studies have been successful in identifying PmP in regional earthquake wavefields using reciprocity to evaluate P-PmP moveout using multiple events (*7*). Our technique is similar; however, it has the added benefit of removing the uncertainty associated with potential errors in the source location and depth. A related verification of our observations comes from the fact that a global inversion for the sensitive parameters of Moho depth and upper mantle velocity under the assumption that our observations were PmP produced results that are highly consistent with previous studies for the region made using independent methods (*36*–*38*). The regional variability, in terms of both absolute and relative values, of Moho depth is also in close agreement with the expected variability for Southern California resolved using receiver functions (*7*, *9*, *40*).

As shown in Fig. 2, for some arrivals before the triplication point, Pn is expected to arrive between Pg and PmP, and for arrivals after the triplication point, Pg is expected to arrive between Pn and PmP. This may lead to the problem that in some cases, Pn or Pg may be misidentified as PmP. It is highly unlikely that Pn would be misidentified as PmP, as Pn is expectedly much lower amplitude than either Pg or PmP in our synthetic tests (fig. S3). The case of Pg following Pn is more problematic; judging by our synthetic tests, Pg is expected to have comparable amplitude to PmP for a range of source-to-receiver distances beyond the triplication point. However, as shown in Fig. 4, for observations on or shortly beyond the triplication point, the correlograms show no evidence of exceptionally early PmP arrivals, as would be expected if we were misclassifying Pg as PmP. Further, there is little evidence of multiple high-amplitude peaks in the correlograms following Pn, as would be expected if Pg were observable in the coda. There are some places where there are estimates of Moho depth made using Pg-PmP differential times and Pn-PmP differential times that are close together (within 5 km). The difference in Moho depths made using these distinct measurements is small (0.2 ± 1.9 km), supporting our expectation that we are picking Pn-PmP differential times. Supplementary evidence can be found in the fact that the Moho variability computed using PmP following Pn agrees with previous studies, as is made abundantly clear by the consistently resolved deepening of the Moho under the San Bernardino Mountains (*7*, *9*, *11*, *40*) and that the resolved upper mantle velocity is highly consistent with Pn tomography models (*37*, *57*). The step across the Garlock Fault and the shallowing of the Moho near Coso are made using PmP picks that follow a Pg first arrival. We also note that for low-angle reflections, there is expectedly phase distortion (*58*). Relatedly, if the recorded first arriving P phase and PmP originate from different quadrants of the focal mechanism, the polarities of these phases may differ. For simplicity and precision, we pick the maximum positive correlation peak, and we expect errors due to phase distortion or mechanism-related polarity reversals to be small. This expectation is supported by the fact that the dominant frequency content of PmP in this study is high (~4 Hz), as illustrated in the stacked cross-correlation wavelet in fig. S4. The upper bound for error due to these distorting effects (~0.1 s) is thus quite small compared to the differential time differences important in this study, as shown in Figs. 7 and 8.

The depth phases sP and sPmP are also of interest because they can produce amplitudes comparable to PmP and in some cases are expected to arrive at times similar to PmP. As shown in fig. S5, the phase sP can have comparable differential times at certain source-to-receiver distances for specific source depths; however, the moveout of the P-sP differential time is markedly different from P-PmP and can easily be distinguished using the moveout criterion of the PmP selection. Also shown in fig. S5, for very shallow events (<2.5 km), the expected arrival times of sPmP assuming a shallower Moho and PmP assuming a deeper Moho can be similar. This is a concern for a small number of earthquakes in this study with shallow source depths. One such important event is the event whose data are shown in Fig. 8B; we describe the uncertainties associated with the shallow depth of this event subsequently. We also note that SmS is observable in these data. SmS is a uniquely important phase because it is sensitive to shear wave velocities in the lower crust (*59*, *60*), and it often produces the strongest ground shaking at regional distances (*61*, *62*). We show an example of an SmS observation next to a PmP observation for one of the earthquake wavefields used in this study in fig. S6. Picking and identifying first S arrivals at regional distances is often challenging, and the SmS cross-correlation peaks are generally not as clear as those of PmP. We thus reserve the systematic identification and analysis of SmS phase differential times for future work.

An important result of this study is that PmP is commonly observable in DAS data; however, we also found that PmP is more commonly not observable. The fact that PmP is observable in some seismograms but not others has been extensively documented (*18*), but the reason for this observation has not been conclusively determined. We do not attempt to resolve this question in this study, but we evaluate how several source and array parameters affect the observability of PmP (fig. S7). We found strong positive correlations between source magnitude and source-to-receiver distance with PmP observability (fig. S7, A and C), and a weak positive correlation between source depth and PmP observability (fig. S7B). Additionally, for the sources for which there was a focal mechanism solution, we computed the minimum angular distance between the expected takeoff ray of PmP and the nearest nodal plane to the observability of PmP (fig. S7E). As expected, we see a positive correlation between these two parameters, suggesting that PmP observability is partially a source effect. Additionally, since DAS is a uniaxial strainmeter, the array geometry may play a role in the observability of PmP. To determine the strength of this effect, we compare the angle between the expected incidence path of PmP and the local array geometry to the observability of PmP (fig. S7D). We find a negative correlation between these two parameters, which is consistent with the expected broadside insensitivity of DAS. The combination of source effect and array geometry produces a stronger correlation (fig. S7F). None of these parameters completely explain the common lack of observability of PmP. There are likely large contributions from parameters that are not so easily tested, such as focusing and defocusing of PmP due to crustal structural variability, energy partitioning due to complex geologic structure (*63*), and the strengthening or weakening of PmP due to differences in the sharpness of the Moho (*64*).

Here, we are mostly concerned with short-wavelength, relative changes in Moho depth. For these changes, we estimate uncertainty (shown in Fig. 5 and fig. S8) using variability in measured P-PmP differential times (see Materials and Methods). However, the use of fixed crustal and upper mantle velocities to invert for Moho depth leads to uncertainty in the absolute crustal thickness. There are trade-offs between the absolute value of the Moho depth and the velocity model used to infer expected P-PmP differential times. We can also evaluate the potential variability in the resolved absolute crustal thickness values due to uncertainties in the velocity model. The Moho depth values are especially sensitive to changes in the lower crustal velocity and, for Moho depths resolved using P-PmP differential times when Pn is the first arrival, upper mantle velocity. Our fixed crustal model considers a set of one-dimensional (1D) profiles drawn from the community velocity model (CVM) (*65*) at expected bounce point, source, and receiver locations, and the variance of these 1D profiles can be used to estimate the potential broad deviations of the lower crustal velocity within our study area (±0.15 km/s). Additionally, we can perturb our best-fitting upper mantle velocities by a small value (±0.1 km/s) and continue to fit our data reasonably well. We invert for Moho depth along the bounce-point profiles using perturbed values (see Materials and Methods) and plot the results in fig. S9. In general, as shown in fig. S10, the deviations are quite small. Varying the lower crustal velocity produces the most substantial change in absolute value, but this is contained within 5 km and the key features in the relative variability are unchanged. Varying the upper mantle velocity shifts only the measurements that trail a Pn first arrival, and these shifts are within 3 km.

Multiple events are necessary to support the observation of shallowing of the Moho near the CVF, meaning we must carefully consider source uncertainty. The two events that suggest a thin crust near Coso are approximately collocated, with depths of 6.9 and 7.1 km in the Southern California Seismic Network (SCSN) catalog and 7.75 and 7.87 km in a catalog with updated event locations made using waveform cross-correlation (*66*). If this feature was an artifact of incorrect event location, both events would need to be severely mislocated in both catalogs, as removal of this anomaly would require these events to be substantially deeper, approximately 15-km depth. We can estimate the potential error in the depths of the SCSN catalog by plotting a distribution of differences between the depths of the events used in this study in the SCSN catalog and the available depths in the waveform relocated seismicity catalog. The vast majority of these differences are within 3 km, and there is no difference present that would be large enough to negate the shallow Moho anomaly. The profile just south of the anomaly that marks the recovery of the Moho to deeper depths is generated by a shallow event (2.4 km). Making the Moho shallower along this profile would require a much shallower event depth, which is highly unlikely given the already shallow depth. We can systematically evaluate the dependence of our Moho depth estimates on source depth by inverting for Moho depths shifted according to the estimated depth uncertainty illustrated in fig. S11 (−1 km to +3 km). The resultant changes in Moho depth are minor and are plotted in figs. S9 and S10. Finally, although we interpret the weak phase in Fig. 8B to be the diffracted phase, this peak could also be a correlogram artifact or the true PmP phase, with the strong peak corresponding to the depth phase (sPmP). If true, the third possibility would only extend the expected region of shallow depths slightly south, at which point there are several profiles with a variety of source depths that all suggest a roughly uniform Moho depth that is consistent with previous results for the region.

## MATERIALS AND METHODS

### Earthquake selection and quality control

For the recording period between August 2021 and August 2023, the DAS array recorded several hundred M2.5+ earthquakes that we identify using the Southern California Seismic Network catalog. Earthquakes of these magnitudes are sufficiently large to exhibit a consistently observable P phase at regional distances in DAS data, which generally have higher noise than broadband data, and thus, we consider only these earthquakes in this study. Of these earthquakes, we consider all events that have at least one source-to-receiver epicentral distance between 70 and 250 km. The lower bound is selected to ensure that each event analyzed could show observable PmP (i.e., arrives before the *S* wave) over a substantial segment of the fiber and thus may be identified with spatial coherence. This lower bound also increases the likelihood that some observations will be made at postcritical distances and thus are more easily identifiable. The upper bound is an estimate of the source-to-receiver distance at which the signal-to-noise ratio of the earthquake wavefield is too low to identify the phases of interest in this study. After this initial quality control, there are 440 candidate earthquakes distributed throughout Southern California with likely high-quality wavefields and potentially estimable P-phase onsets with which we may analyze the Moho reflected phase.

We apply a straightforward and semi-automated framework for obtaining P-to-PmP differential times via autocorrelation. We first select a window for each of the candidate earthquakes starting 30 s before and ending 90 s after the cataloged origin time and make travel-time picks on these events using PhaseNet DAS (*34*). We then visually evaluate these picks, keeping only first arrival picks that we can confidently attribute to the first arriving P phase. Of the initial set of events, 229 events had visually identifiable P-phase onsets and some preserved first arrival picks. We then compute the expected first arrivals for each event using the HK 1D model (*36*) and the software TauP (*67*) and correct the absolute times of these computed arrivals using the preserved pick times. This workflow provides us with calibrated approximate onset times for every channel for each event. Once we have these onset times, we select a time window for each record that is 4 s before and 8 s after the approximate onset time. We then spectrally whiten the signal and filter the signal between 0.2 and 5 Hz, a filter band that balances maintaining as much of the earthquake spectra as possible while reducing high- and low-frequency noise. We then select a 1-s window around the approximate first arrival (0.3 s before and 0.7 s after) and correlate this time window with the full-time window. This is distinct from other autocorrelation workflows that simply autocorrelate the full-time window (*68*). We make this choice because the first arrivals in these data are often very low amplitude and using this narrow window scales the contribution of the first arriving P phase to make the P-PmP correlation peak identifiable. We then visually inspect each of the autocorrelated wavefields, using P-PmP relative arrival time computed using the HK model as a reference. We manually select spatial windows that encompass high-amplitude, spatially coherent correlation peaks that maintain a reasonable moveout for PmP at the respective source-to-receiver distances. We stipulate that these windows must encompass at least 500 channels, as this is the minimum number we deem necessary to evaluate array-side moveout. From this manual selection, we obtain large spatial windows of PmP observations for 72 events. For each of these spatial windows, we pick P-PmP differential times by selecting the local maxima corresponding to the PmP onset in the autocorrelation profiles. These local maxima are selected using a visually identified time window within which the P-PmP autocorrelation peak is observed. Additionally, by visually evaluating the change in differential time with source-to-receiver distance, we determine for each observation whether PmP is trailing Pg or Pn. A decrease in the differential time with distance corresponds to PmP trailing Pg, and the opposite is the case for PmP trailing Pn. Using this workflow, we select over 200,000 accurate and precise P-PmP differential times shown in Fig. 4.

### Inverting for upper mantle velocity and crustal thickness variability

Here, we fix the crustal velocity according to an ensemble of profiles from the CVM (*65*). These profiles are drawn from the source, bounce-point, and channel locations used in this study. We reduce these models for comparability and simplicity by depth-averaging the upper (0 to 5.5 km), middle (5.5 to 16 km), and lower (16 km to Moho) crustal velocities to generate three-layer models and take a representative model as our fixed crustal model. We then perform a grid search for a representative crustal thickness and upper mantle velocity for our entire dataset. We bootstrap by randomly sampling which events we use in the inversion to estimate the error. For each P-PmP differential time observation, we compute an expected differential time using TauP for a set of Moho depth and upper mantle velocity pairs, and we determine the pair that minimizes the misfit to all our observations. We find that the optimal pair is a Moho depth of 31.5 km and an upper mantle velocity of 8.1 km/s. We then use our fixed crustal and optimal upper mantle velocities and invert for crustal thickness along each of our bounce-point profiles. For each source-receiver pair, we compute expected P-PmP differential times for a suite of Moho depths and determine which Moho depths minimize the misfit to the data for 100 sample segments (1 km) along the fiber; this segment length is selected to improve the stability of the inversion and is shorter than the minimum wavelength in our data. We estimate the uncertainty due to errors in the differential travel-time measurements by computing the standard deviation of the 100 sample segments and estimating Moho depth for differential times that are ±2σ from the mean. This uncertainty is shown for key profiles in Fig. 5 and for the entire study area in fig. S8. The loss is evaluated as the L2 norm. Additionally, since we have observations of whether our PmP phase trails a Pg or Pn first arrival, we penalize any mismatch between our observed first arrival and the modeled first arrivals. To accomplish this, we multiply the loss by $\frac{n}{m+\epsilon }$, where *n* is the number of observations within a segment, *m* is the number of observations for which the observed and the expected first arrivals match, and ϵ is a small number incorporated to prevent singularities. Once we have a best-fitting solution, we compute the expected bounce-point locations using the source location, velocity model, and best-fitting Moho depth to determine the location of the Moho depth measurement.

### Performing synthetic tests

To generate the synthetic results shown in Fig. 6, we use the software Salvus (*69*), which uses the spectral element method to simulate wavefield propagation. We parameterize the velocity model using our 1D model derived from a sampling of the CVM and our inverted upper mantle velocity for the region, and we set the Moho depth to be 30 km. We smooth the a priori defined layer boundaries within our 1D model to minimize artifact reflections in the synthetics. We parameterize our model as a 60-km segment of strainmeters at the surface of our model and emplace an isotropic source with an impulsive source time function at 10-km depth and 55-km lateral distance from the first station. The step we impose in the Moho is 5 km high and has a 20-km plateau with 5-km linear ramps on either side of the step. The high velocity zone imposed in the lower crust is a 7.5% velocity increase with 20-km width. The low-velocity zone imposed in the middle crust is a 9% velocity reduction with 20-km width. These velocity anomalies have smoothed edges imposed using a Gaussian filter with a standard deviation of 250 m. The expected arrival times of P and PmP are computed using TauP (*67*), and the 1D model is used to parameterize the synthetic with an unmodified Moho structure. The plotted synthetic autocorrelations are produced using the processing described above, and we use the computed P times as our first arrival times for the autocorrelation. Animations for these synthetic tests are shown in movie S1.

## References

[1] D. McKenzie, F. Nimmo, J. A. Jackson, P. B. Gans, E. L. Miller, Characteristics and consequences of flow in the lower crust. J. Geophys. Res. Solid Earth 105, 11029–11046 (2000).

[2] L. Zhu, B. J. Mitchell, N. Akyol, I. Cemen, K. Kekovali, Crustal thickness variations in the Aegean region and implications for the extension of continental crust. J. Geophys. Res. Solid Earth 111, 2005JB003770 (2006).

[3] Z. Liu, X. Tian, R. Gao, G. Wang, Z. Wu, B. Zhou, P. Tan, S. Nie, G. Yu, G. Zhu, X. Xu, New images of the crustal structure beneath eastern Tibet from a high-density seismic array. Earth Planet. Sci. Lett. 480, 33–41 (2017).

[4] Y. Luo, M. D. Long, P. Karabinos, Y. D. Kuiper, S. Rondenay, J. C. Aragon, L. Sawade, P. Makus, High-resolution Ps receiver function imaging of the crust and mantle lithosphere beneath southern new England and tectonic implications. J. Geophys. Res. Solid Earth 126, e2021JB022170 (2021).

[5] C. Yu, J. C. Castellanos, Z. Zhan, Imaging strong lateral heterogeneities across the contiguous US using body-to-surface wave scattering. J. Geophys. Res. Solid Earth 126, e2020JB020798 (2021).

[6] L. Zhu, Crustal structure across the San Andreas Fault, Southern California from teleseismic converted waves. Earth Planet. Sci. Lett. 179, 183–190 (2000).

[7] T. Li, J. Yao, S. Wu, M. Xu, P. Tong, Moho complexity in Southern California revealed by local PmP and teleseismic Ps waves. J. Geophys. Res. Solid Earth 127, e2021JB023033 (2022).

[8] S. Sui, W. Shen, W. Holt, J. Kim, Crustal architecture across Southern California and its implications on San Andreas fault development. Geophys. Res. Lett. 50, e2022GL101976 (2023).

[9] Z. Yan, R. W. Clayton, Regional mapping of the crustal structure in Southern California from receiver functions. J. Geophys. Res. Solid Earth 112, 2006JB004622 (2007).

[10] M. J. Cheadle, B. L. Czuchra, T. Byrne, C. J. Ando, J. E. Oliver, L. D. Brown, S. Kaufman, P. E. Malin, R. A. Phinney, The deep crustal structure of the Mojave Desert, California, from Cocorp seismic reflection data. Tectonics 5, 293–320 (1986).

[11] Y.-G. Li, T. L. Henyey, P. C. Leary, Seismic reflection constraints on the structure of the crust beneath the San Bernardino Mountains, Transverse Ranges, Southern California. J. Geophys. Res. Solid Earth 97, 8817–8830 (1992).

[12] D. N. Steer, J. H. Knapp, L. D. Brown, H. P. Echtler, D. L. Brown, R. Berzin, Deep structure of the continental lithosphere in an unextended orogen: An explosive-source seismic reflection profile in the Urals (Urals Seismic Experiment and Integrated Studies (URSEIS 1995)). Tectonics 17, 143–157 (1998).

[13] K. K. Davenport, J. A. Hole, B. Tikoff, R. M. Russo, S. H. Harder, A strong contrast in crustal architecture from accreted terranes to craton, constrained by controlled-source seismic data in Idaho and eastern Oregon. Lithosphere 9, 325–340 (2017).

[14] M. M. Fliedner, S. L. Klemperer, N. I. Christensen, Three-dimensional seismic model of the Sierra Nevada arc, California, and its implications for crustal and upper mantle composition. J. Geophys. Res. Solid Earth 105, 10899–10921 (2000).

[15] R. E. Marzen, D. J. Shillington, D. Lizarralde, S. H. Harder, Constraints on appalachian orogenesis and continental rifting in the Southeastern United States from wide-angle seismic data. J. Geophys. Res. Solid Earth 124, 6625–6652 (2019).

[16] L. L. Worthington, K. C. Miller, E. A. Erslev, M. L. Anderson, K. R. Chamberlain, A. F. Sheehan, W. L. Yeck, S. H. Harder, C. S. Siddoway, Crustal structure of the Bighorn Mountains region: Precambrian influence on Laramide shortening and uplift in north-central Wyoming. Tectonics 35, 208–236 (2016).

[17] Z. Zhang, S. L. Klemperer, West-east variation in crustal thickness in northern Lhasa block, central Tibet, from deep seismic sounding data. J. Geophys. Res. Solid Earth 110, B09403 (2005).

[18] W. Ding, T. Li, X. Yang, K. Ren, P. Tong, Deep neural networks for creating reliable PmP database with a case study in Southern California. J. Geophys. Res. Solid Earth 127, e2021JB023830 (2022).

[19] J. Nakajima, T. Matsuzawa, A. Hasegawa, Moho depth variation in the central part of northeastern Japan estimated from reflected and converted waves. Phys. Earth Planet. Inter. 130, 31–47 (2002).

[20] K. B. Richards-Dinger, P. M. Shearer, Estimating crustal thickness in Southern California by stacking *PmP* arrivals. J. Geophys. Res. Solid Earth 102, 15211–15224 (1997).

[21] M. K. Salah, D. Zhao, Mapping the crustal thickness in southwest Japan using Moho-reflected waves. Phys. Earth Planet. Inter. 141, 79–94 (2004).

[22] X. Huang, D. Yang, P. Tong, J. Badal, Q. Liu, Wave equation-based reflection tomography of the 1992 Landers earthquake area. Geophys. Res. Lett. 43, 1884–1892 (2016).

[23] T. Li, S. Wu, P. Tong, Multilevel transcrustal magmatic system beneath the Geysers-Clear Lake area. Proc. Natl. Acad. Sci. U.S.A. 121, e2317809121 (2024). 38466842 10.1073/pnas.2317809121 PMC10962962

[24] D. Wang, S. Wu, T. Li, P. Tong, Y. Gao, Elongated magma plumbing system beneath the Coso Volcanic Field, California, Constrained by seismic reflection tomography. J. Geophys. Res. Solid Earth 127, e2021JB023582 (2022).

[25] S. Xia, D. Zhao, X. Qiu, J. Nakajima, T. Matsuzawa, A. Hasegawa, Mapping the crustal structure under active volcanoes in central Tohoku, Japan using P and PmP data. Geophys. Res. Lett. 34, 2007GL030026 (2007).

[26] Z. Zhan, Distributed acoustic sensing turns fiber-optic cables into sensitive seismic antennas. Seismol. Res. Lett. 91, 1–15 (2020).

[27] F. Cheng, B. Chi, N. J. Lindsey, T. C. Dawe, J. B. Ajo-Franklin, Utilizing distributed acoustic sensing and ocean bottom fiber optic cables for submarine structural characterization. Sci. Rep. 11, 5613 (2021). 33692381 10.1038/s41598-021-84845-y PMC7946901

[28] Z. J. Spica, M. Perton, E. R. Martin, G. C. Beroza, B. Biondi, Urban seismic site characterization by fiber-optic seismology. J. Geophys. Res. Solid Earth 125, e2019JB018656 (2020).

[29] Y. Yang, J. W. Atterholt, Z. Shen, J. B. Muir, E. F. Williams, Z. Zhan, Sub-kilometer correlation between near-surface structure and ground motion measured with distributed acoustic sensing. Geophys. Res. Lett. 49, e2021GL096503 (2022).

[30] E. Biondi, W. Zhu, J. Li, E. F. Williams, Z. Zhan, An upper-crust lid over the Long Valley magma chamber. Sci. Adv. 9, eadi9878 (2023). 37851798 10.1126/sciadv.adi9878 PMC10584340

[31] C. Yu, Z. Zhan, N. J. Lindsey, J. B. Ajo-Franklin, M. Robertson, The potential of DAS in teleseismic studies: Insights from the Goldstone experiment. Geophys. Res. Lett. 46, 1320–1328 (2019).

[32] R. Fernández-Ruiz, M., Costa, L. & F. Martins, H., Distributed acoustic sensing using chirped-pulse phase-sensitive OTDR technology. Sensors 19, 4368 (2019). 31601056 10.3390/s19204368 PMC6832391

[33] J. Atterholt, Z. Zhan, Y. Yang, Fault zone imaging with distributed acoustic sensing: Body-to-surface wave scattering. J. Geophys. Res. Solid Earth 127, e2022JB025052 (2022).

[34] W. Zhu, E. Biondi, J. Li, J. Yin, Z. E. Ross, Z. Zhan, Seismic arrival-time picking on distributed acoustic sensing data using semi-supervised learning. Nat. Commun. 14, 8192 (2023). 38081845 10.1038/s41467-023-43355-3 PMC10713581

[35] N. J. Lindsey, T. C. Dawe, J. B. Ajo-Franklin, Illuminating seafloor faults and ocean dynamics with dark fiber distributed acoustic sensing. Science 366, 1103–1107 (2019). 31780553 10.1126/science.aay5881

[36] D. Hadley, H. Kanamori, Seismic structure of the transverse ranges, California. Geol. Soc. Am. Bull. 88, 1469 (1977).

[37] J. S. Buehler, P. M. Shearer, Anisotropy and *Vp*/*Vs* in the uppermost mantle beneath the western United States from joint analysis of *Pn* and *Sn* phases. J. Geophys. Res. Solid Earth 119, 1200–1219 (2014).

[38] C. Tape, A. Plesch, J. H. Shaw, H. Gilbert, Estimating a continuous Moho surface for the California unified velocity model. Seismol. Res. Lett. 83, 728–735 (2012).

[39] S. A. Raikes, Regional variations in upper mantle structure beneath Southern California. Geophys. J. Int. 63, 187–216 (1980).

[40] L. Zhu, H. Kanamori, Moho depth variation in Southern California from teleseismic receiver functions. J. Geophys. Res. Solid Earth 105, 2969–2980 (2000).

[41] J. N. Louie, J. Qin, Subsurface imaging of the Garlock Fault, Cantil Valley, California. J. Geophys. Res. Solid Earth 96, 14461–14479 (1991).

[42] B. Wernicke, J. E. Spencer, B. C. Burchfiel, P. L. Guth, Magnitude of crustal extension in the southern Great Basin. Geology 10, 499–502 (1982).

[43] I. W. Bailey, Y. Ben-Zion, T. W. Becker, M. Holschneider, Quantifying focal mechanism heterogeneity for fault zones in central and Southern California. Geophys. J. Int. 183, 433–450 (2010).

[44] H. Qiu, B. Chi, Y. Ben-Zion, Internal structure of the central Garlock Fault zone from ridgecrest aftershocks recorded by dense linear seismic arrays. Geophys. Res. Lett. 50, e2022GL101761 (2023).

[45] J. Atterholt, Z. Zhan, Y. Yang, W. Zhu, Imaging the Garlock Fault zone with a fiber: A limited damage zone and hidden bimaterial contrast. J. Geophys. Res. Solid Earth 129, e2024JB028900 (2024).

[46] P. Molnar, H. J. Anderson, E. Audoine, D. Eberhart-Phillips, K. R. Gledhill, E. R. Klosko, T. V. McEvilly, D. Okaya, M. K. Savage, T. Stern, F. T. Wu, Continuous deformation versus faulting through the continental lithosphere of New Zealand. Science 286, 516–519 (1999). 10521344 10.1126/science.286.5439.516

[47] R. Y. Chuang, K. M. Johnson, Reconciling geologic and geodetic model fault slip-rate discrepancies in Southern California: Consideration of nonsteady mantle flow and lower crustal fault creep. Geology 39, 627–630 (2011).

[48] A. A. Allam, V. Schulte-Pelkum, Y. Ben-Zion, C. Tape, N. Ruppert, Z. E. Ross, Ten kilometer vertical Moho offset and shallow velocity contrast along the Denali fault zone from double-difference tomography, receiver functions, and fault zone head waves. Tectonophysics 721, 56–69 (2017).

[49] J. Saleeby, M. Ducea, D. Clemens-Knott, Production and loss of high-density batholithic root, Southern Sierra Nevada, California. Tectonics 22, 2002TC001374 (2003).

[50] G. Zandt, H. Gilbert, T. J. Owens, M. Ducea, J. Saleeby, C. H. Jones, Active foundering of a continental arc root beneath the southern Sierra Nevada in California. Nature 431, 41–46 (2004). 15343326 10.1038/nature02847

[51] S. L. Dougherty, C. Jiang, R. W. Clayton, B. Schmandt, S. M. Hansen, Seismic evidence for a fossil slab origin for the Isabella anomaly. Geophys. J. Int. 224, 1188–1196 (2020).

[52] Y. Wang, D. W. Forsyth, C. J. Rau, N. Carriero, B. Schmandt, J. B. Gaherty, B. Savage, Fossil slabs attached to unsubducted fragments of the Farallon plate. Proc. Natl. Acad. Sci. U.S.A. 110, 5342–5346 (2013). 23509274 10.1073/pnas.1214880110 PMC3619369

[53] M. V. Bernardino, C. H. Jones, W. Levandowski, I. Bastow, T. J. Owens, G. Hersh, A multicomponent Isabella anomaly: Resolving the physical state of the Sierra Nevada upper mantle from Vp/Vs anisotropy tomography. Geosphere 15, 2018–2042 (2019).

[54] C. Jiang, B. Schmandt, S. M. Hansen, S. L. Dougherty, R. W. Clayton, J. Farrell, F.-C. Lin, Rayleigh and S wave tomography constraints on subduction termination and lithospheric foundering in central California. Earth Planet. Sci. Lett. 488, 14–26 (2018).

[55] Q. Zhang, G. Lin, Three-dimensional Vp and Vp/Vs models in the Coso geothermal area, California: Seismic characterization of the magmatic system. J. Geophys. Res. Solid Earth 119, 4907–4922 (2014).

[56] M. Grad, T. Tiira, ESC Working Group, The Moho depth map of the European Plate. Geophys. J. Int. 176, 279–292 (2009).

[57] J. S. Buehler, P. M. Shearer, Pn tomography of the western United States using USArray. J. Geophys. Res. Solid Earth 115, 2009JB006874 (2010).

[58] K. Aki, P. G. Richards, *Quantitative Seismology* (University Science Books, 2002).

[59] I. Kay, G. Musacchio, D. White, I. Asudeh, B. Roberts, D. Forsyth, Z. Hajnal, B. Koperwhats, D. Farrell, Imaging the moho and *V_p_/V_s_* ratio in the Western Superior Archean Craton with wide angle reflections. Geophys. Res. Lett. 26, 2585–2588 (1999).

[60] Z. Zhan, S. Ni, D. V. Helmberger, R. W. Clayton, Retrieval of Moho-reflected shear wave arrivals from ambient seismic noise. Geophys. J. Int. 182, 408–420 (2010).

[61] K.-S. Liu, Y.-B. Tsai, Large effects of moho reflections (SmS) on peak ground motion in Northwestern Taiwan. Bull. Seismol. Soc. Am. 99, 255–267 (2009).

[62] P. Somerville, J. Yoshimura, The influence of critical Moho Reflections on strong ground motions recorded in San Francisco and Oakland during the 1989 Loma Prieta Earthquake. Geophys. Res. Lett. 17, 1203–1206 (1990).

[63] J. Mori, D. Helmberger, Large-amplitude Moho reflections ( *SmS* ) from Landers aftershocks, Southern California. Bull. Seismol. Soc. Am. 86, 1845–1852 (1996).

[64] V. Levin, J. A. VanTongeren, A. Servali, How sharp is the sharp Archean Moho? Example from eastern superior province. Geophys. Res. Lett. 43, 1928–1933 (2016).

[65] E.-J. Lee, P. Chen, T. H. Jordan, P. B. Maechling, M. A. M. Denolle, G. C. Beroza, Full-3-D tomography for crustal structure in Southern California based on the scattering-integral and the adjoint-wavefield methods. J. Geophys. Res. Solid Earth 119, 6421–6451 (2014).

[66] E. Hauksson, W. Yang, P. M. Shearer, Waveform relocated earthquake catalog for Southern California (1981 to June 2011). Bull. Seismol. Soc. Am. 102, 2239–2244 (2012).

[67] H. P. Crotwell, T. J. Owens, J. Ritsema, The TauP Toolkit: Flexible seismic travel-time and ray-path utilities. Seismol. Res. Lett. 70, 154–160 (1999).

[68] J. R. Delph, A. Levander, F. Niu, Constraining crustal properties using receiver functions and the autocorrelation of earthquake-generated body waves. J. Geophys. Res. Solid Earth 124, 8981–8997 (2019).

[69] M. Afanasiev, C. Boehm, M. van Driel, L. Krischer, M. Rietmann, D. A. May, M. G. Knepley, A. Fichtner, Modular and flexible spectral-element waveform modelling in two and three dimensions. Geophys. J. Int. 216, 1675–1692 (2019).

