# Supplementary material for: Fine-scale Southern California Moho structure uncovered with distributed acoustic sensing
Source: Sci Adv. 2024 Nov 27;10(48):eadr3327. doi: 10.1126/sciadv.adr3327 (PMC11601198; doi:10.1126/sciadv.adr3327)
Supplement: Supplementary file 1 — Figs. S1 to S11 [file sciadv.adr3327_sm.pdf]

Science Advances
AAAS

Supplementary Materials for

Fine-scale Southern California Moho structure uncovered with distributed acoustic sensing

James Atterholt and Zhongwen Zhan

Corresponding author: James Atterholt, jatterholt@usgs.gov

Sci. Adv. **10**, eadr3327 (2024)
DOI: 10.1126/sciadv.adr3327

The PDF file includes:

Figs. S1 to S11

Other Supplementary Material for this manuscript includes the following:

Movie S1

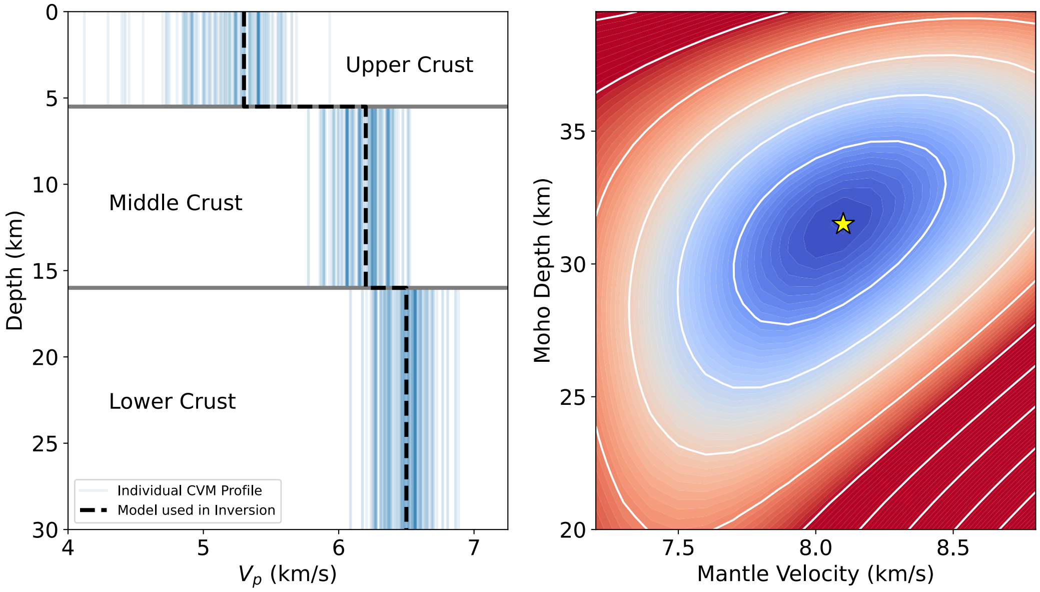

Fig. S1.

Left: Ensemble of crustal models drawn from the Community Velocity Model. Light blue lines are each a profile included in the ensemble. Black dotted line is the representative model used throughout this study. Right: Global Moho depth and upper mantle velocity loss distribution described in the main text. Red and blue indicate higher and lower loss respectively. Yellow star indicates best fitting solution.

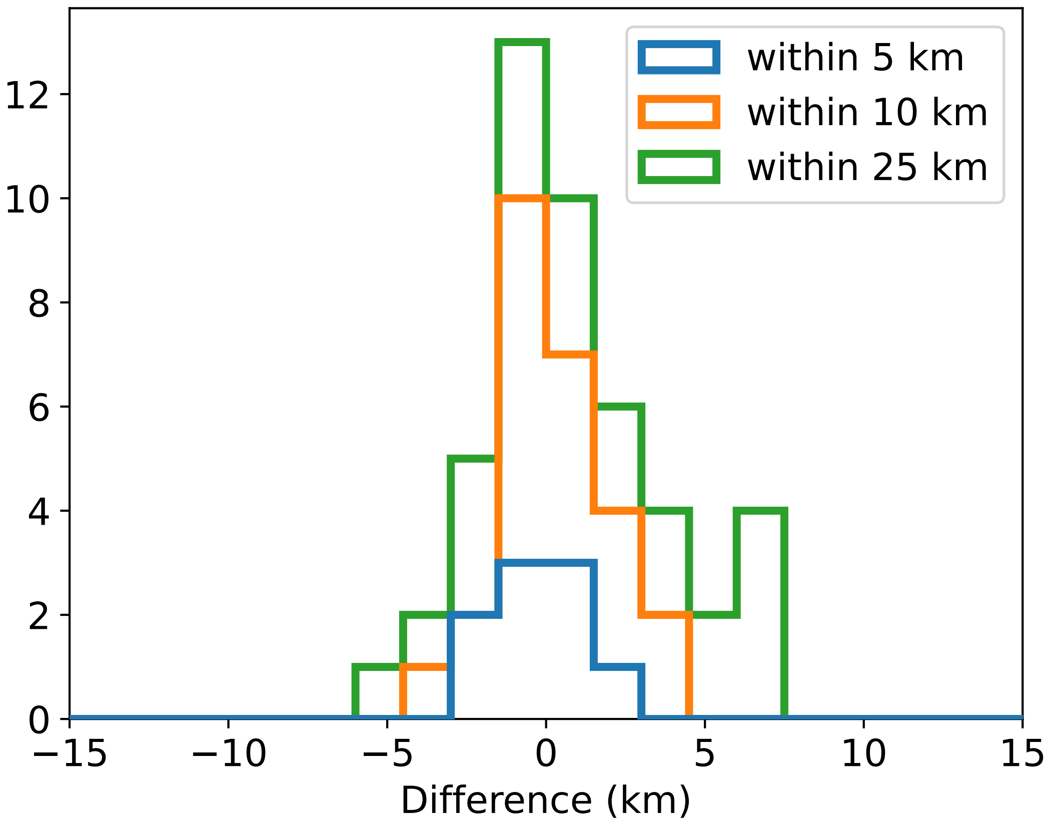

Fig. S2.

Histogram of differences between nearby Moho depth estimates made in this study and those of a prior receiver function study[9]. Different colors correspond to different maximum distances between measurement points used as prerequisites for inclusion in the histogram. Measurements from receiver functions[9] are included only once using the nearest measurement from this study.

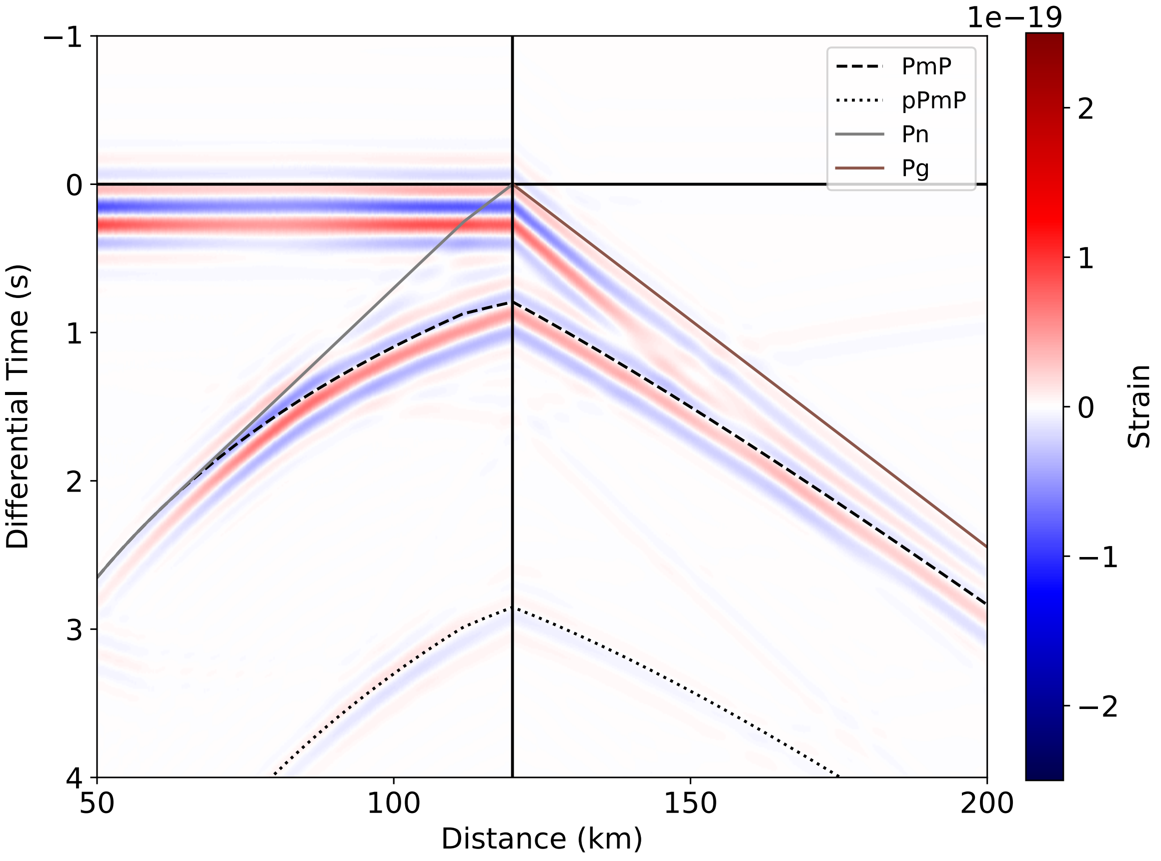

Fig. S3.

Synthetic wavefield generated using the same parameters as the flat Moho model in Figure 6, but with an extended measuring array.

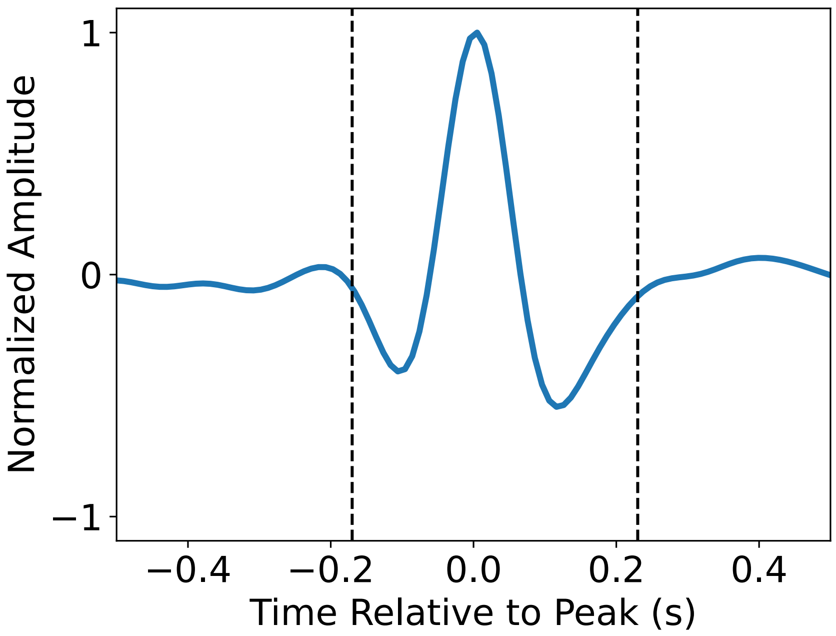

Fig. S4. Full stack of all cross-correlation wavelets associated with PmP used in this study. Alignment is performed using the peak picks shown in Figure 4. Black dotted lines encompass 1.5 wavelengths.

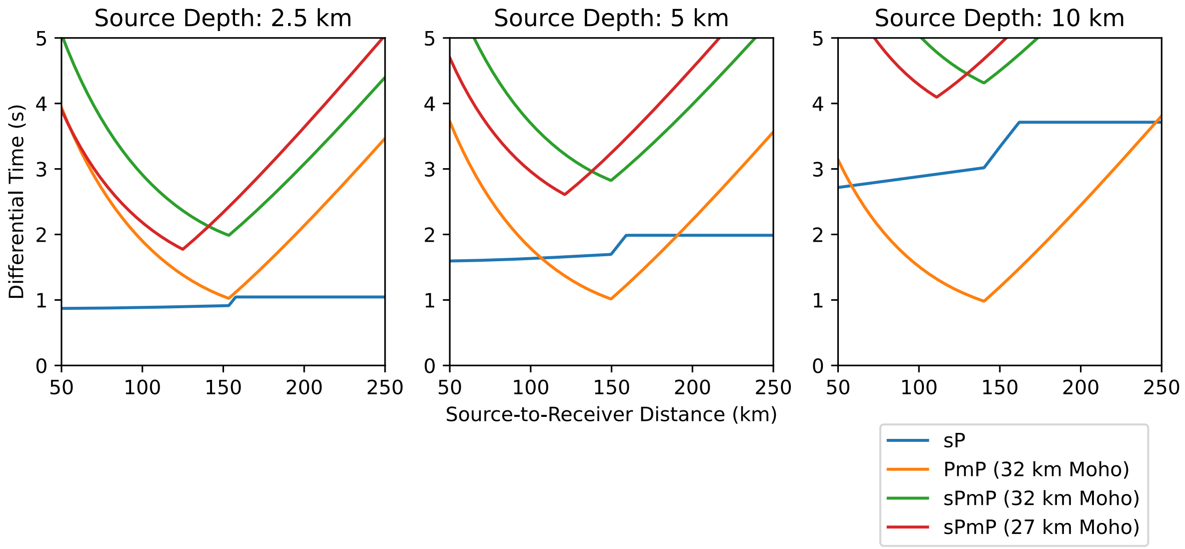

Fig. S5. Phase-P differential times for a diversity of source depth, Moho depth, and source-to-receiver distance combinations.

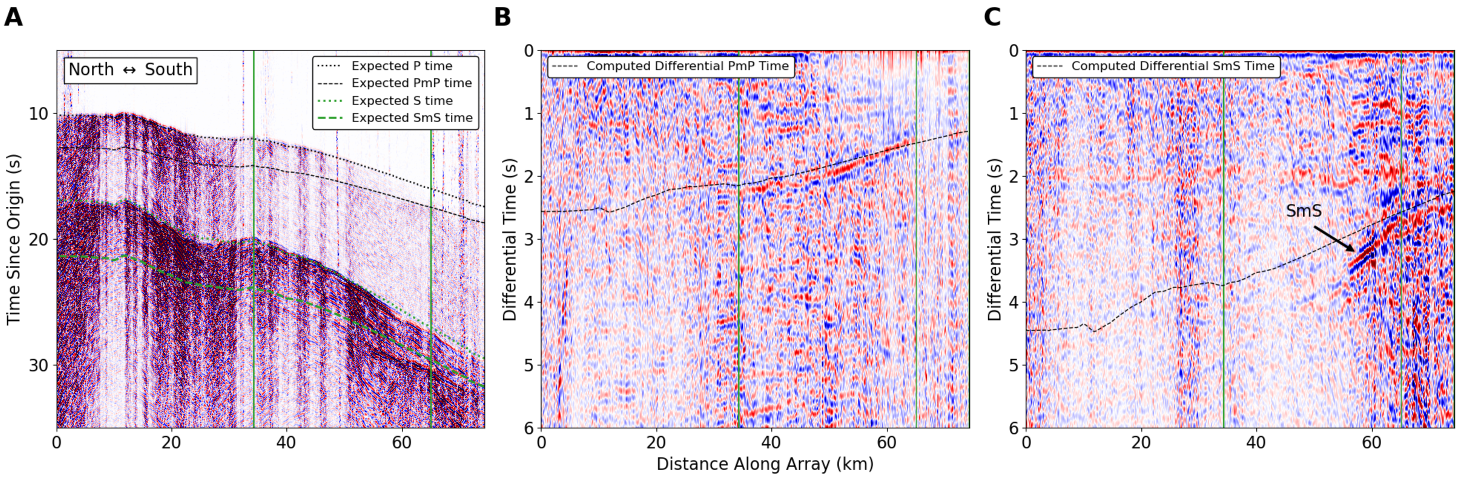

Fig. S6. Example of an SmS observation made using one of the earthquakes in this study. Autocorrelation for SmS was produced by correlating a time window around the earthquake S-wave arrival (0.5 s before to 1.0 s after) with the S-wave coda. Earthquake location is indicated with a diamond marker in Figure 1. Vertical green lines mark the bounds over which PmP is observable.

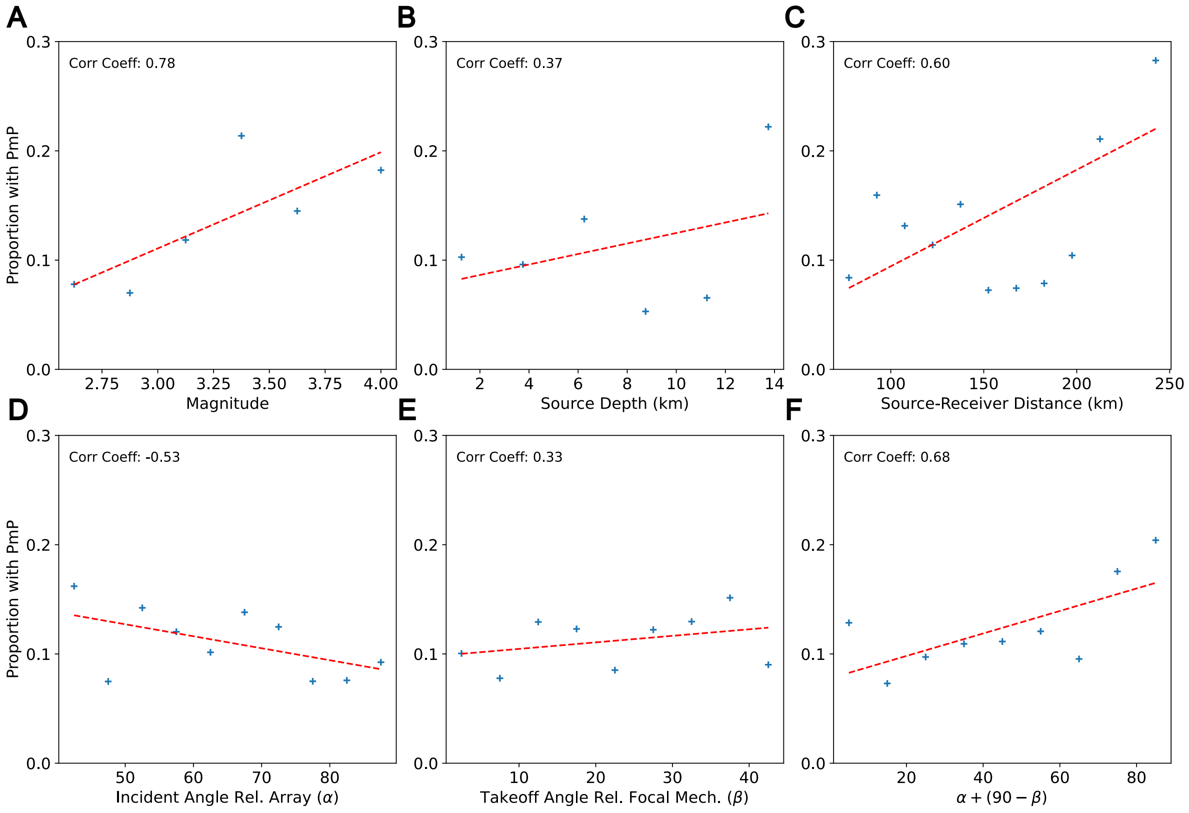

Fig. S7.

Comparison of several parameters with the proportion of station-channel pairs for which PmP was observable in this study.

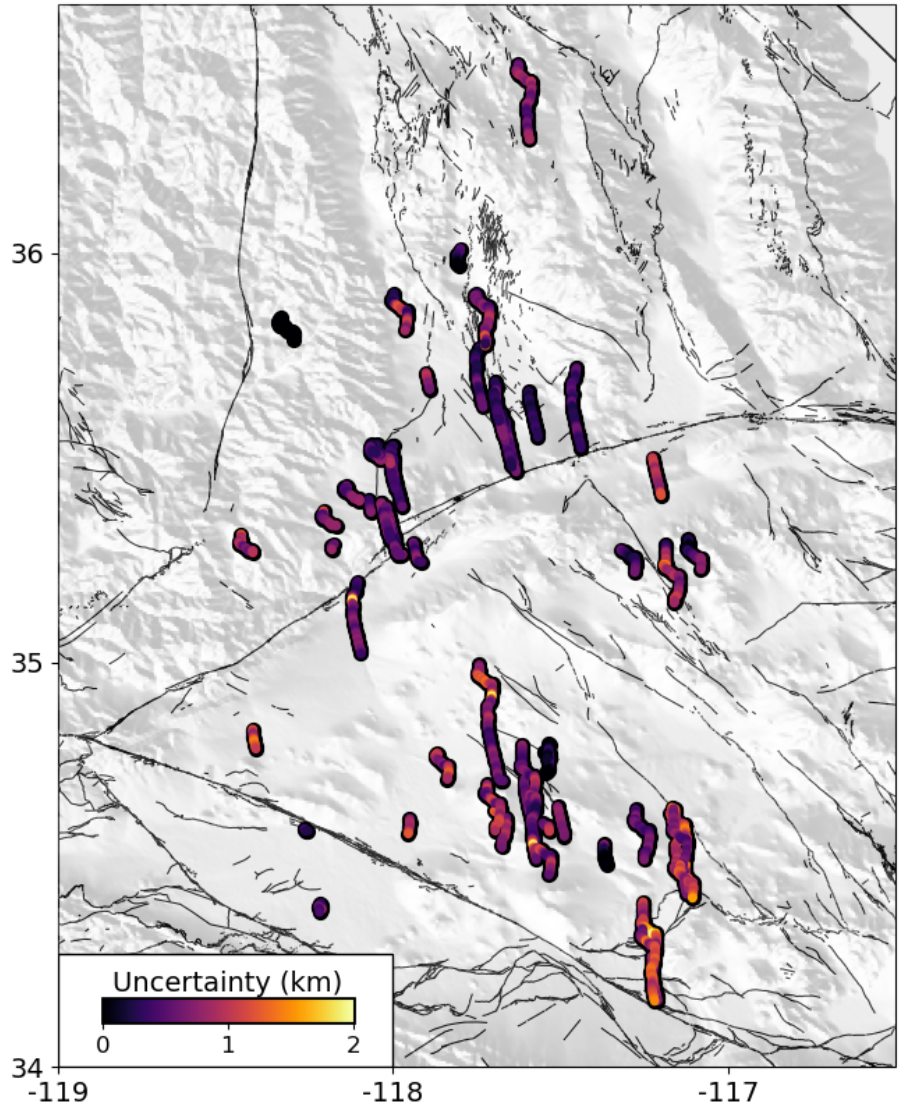

Fig. S8.
Estimates of uncertainty of Moho depth values computed from variability in P-PmP differential time picks.

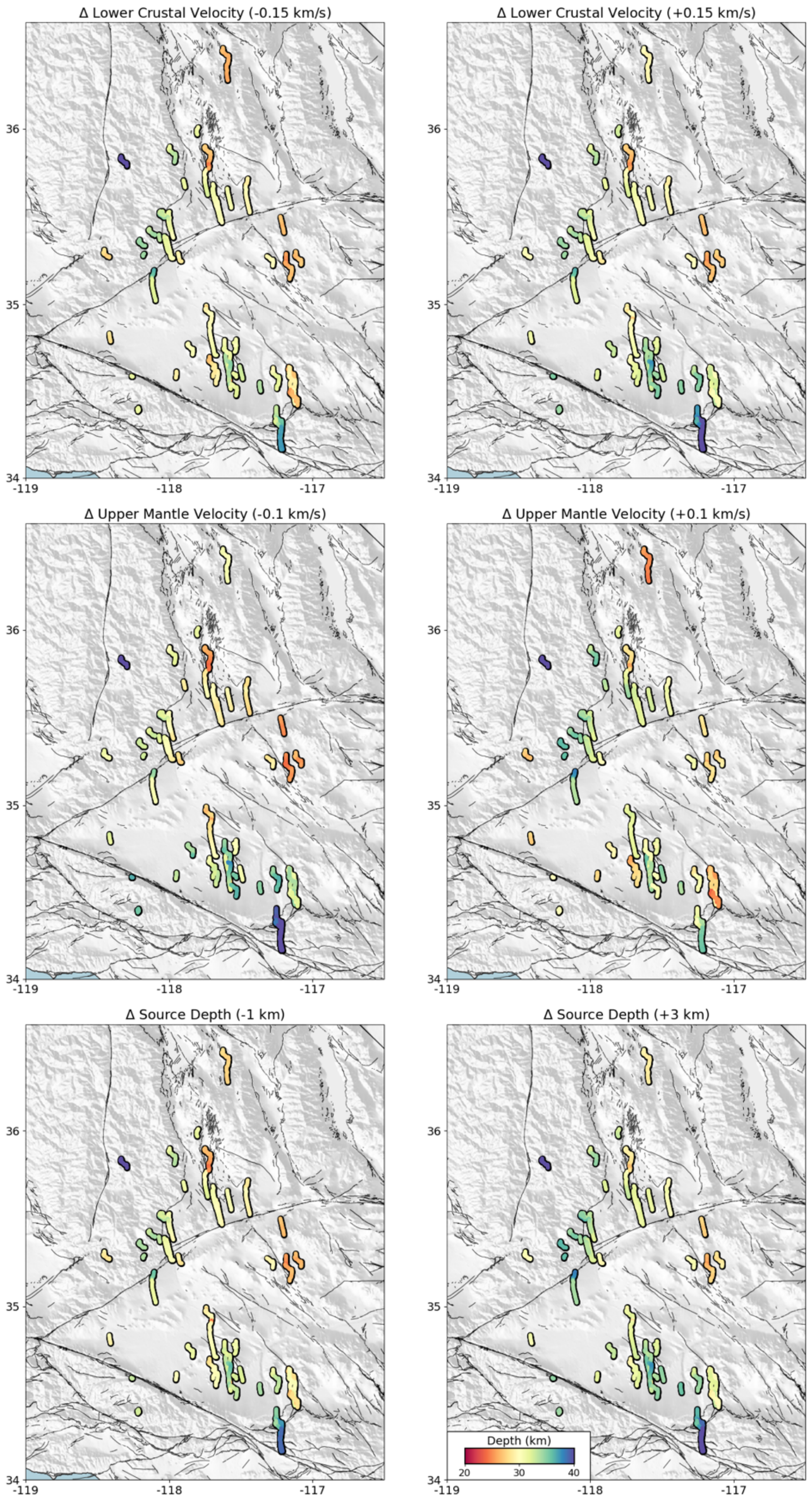

Fig. S9.

Moho depth distributions computed using modified velocity model parameters and shifted source depths.

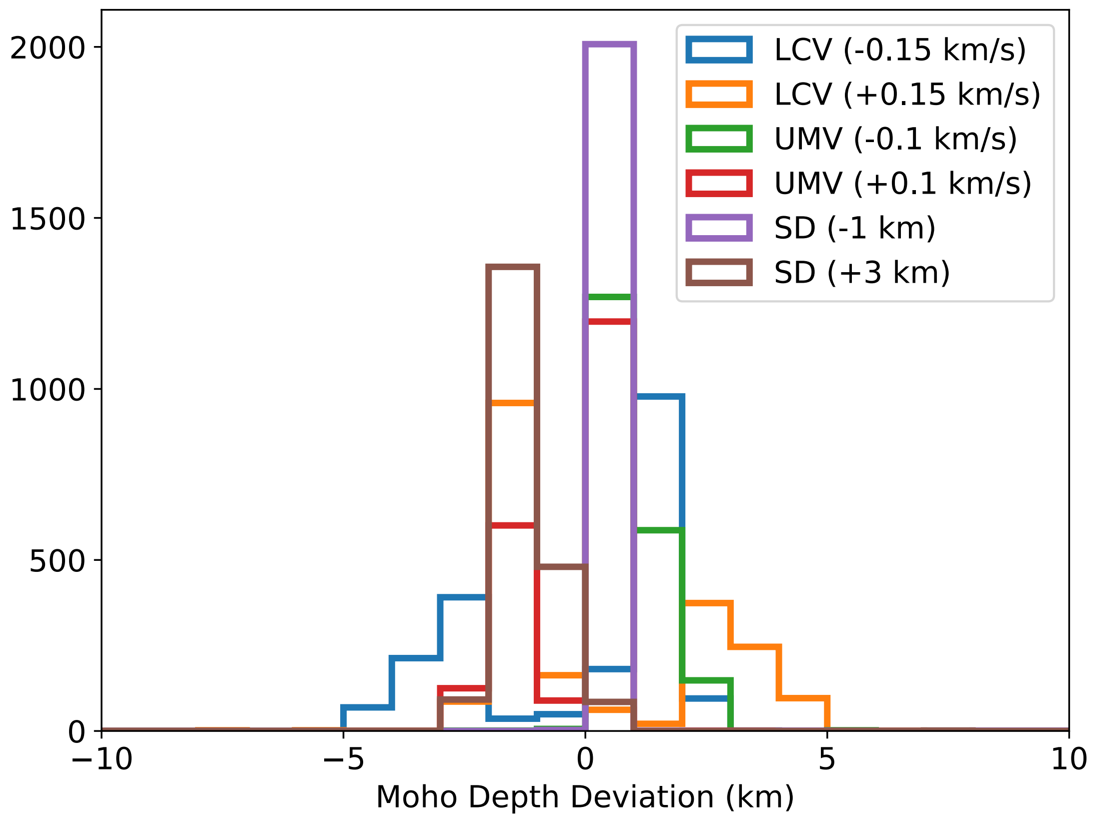

Fig. S10.

Distribution of differences between Moho depth values determined using the perturbations shown in Figure S8 and those of the preferred model shown in Figure 5.

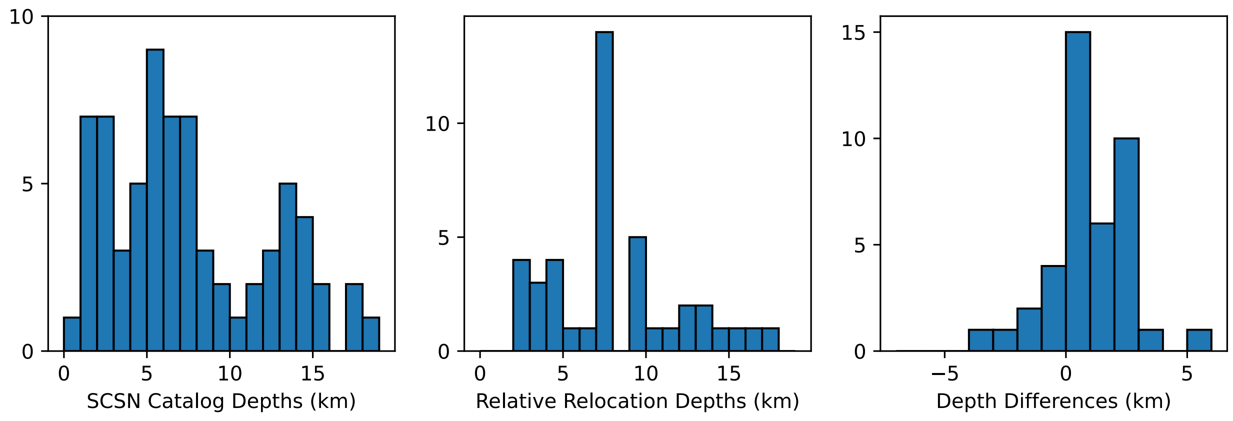

Fig. S11.

Distribution of depths for the SCSN catalog and waveform cross-correlation relocation catalog[66] for events used in the study and the distribution of depth differences between catalogs.
